# Will "Combined Prevention" Eliminate Racial/Ethnic Disparities in HIV Infection among Persons Who Inject Drugs in New York City?

**DOI:** 10.1371/journal.pone.0126180

**Published:** 2015-05-12

**Authors:** Don Des Jarlais, Kamyar Arasteh, Courtney McKnight, Jonathan Feelemyer, Holly Hagan, Hannah Cooper, Aimee Campbell, Susan Tross, David Perlman

**Affiliations:** 1 The Baron Edmond de Rothschild Chemical Dependency Institute, Mount Sinai Beth Israel, New York, New York, United States of America; 2 College of Nursing, New York University, New York, New York, United States of America; 3 Rollins School of Public Health at Emory University, Atlanta, Georgia, United States of America; 4 Department of Psychiatry, Columbia University, New York, New York, United States of America; University of Nebraska Medical Center, UNITED STATES

## Abstract

It has not been determined whether implementation of combined prevention programming for persons who inject drugs reduce racial/ethnic disparities in HIV infection. We examine racial/ethnic disparities in New York City among persons who inject drugs after implementation of the New York City Condom Social Marketing Program in 2007. Quantitative interviews and HIV testing were conducted among persons who inject drugs entering Mount Sinai Beth Israel drug treatment (2007–2014). 703 persons who inject drugs who began injecting after implementation of large-scale syringe exchange were included in the analyses. Factors independently associated with being HIV seropositive were identified and a published model was used to estimate HIV infections due to sexual transmission. Overall HIV prevalence was 4%; Whites 1%, African-Americans 17%, and Hispanics 4%. Adjusted odds ratios were 21.0 (95% CI 5.7, 77.5) for African-Americans to Whites and 4.5 (95% CI 1.3, 16.3) for Hispanics to Whites. There was an overall significant trend towards reduced HIV prevalence over time (adjusted odd ratio = 0.7 per year, 95% confidence interval (0.6–0.8). An estimated 75% or more of the HIV infections were due to sexual transmission. Racial/ethnic disparities among persons who inject drugs were not significantly different from previous disparities. Reducing these persistent disparities may require new interventions (treatment as prevention, pre-exposure prophylaxis) for all racial/ethnic groups.

## Introduction

Significant racial/ethnic disparities in HIV infection among persons who inject drugs (PWID) have been observed in many countries, with ethnic minority group members [1] and females [2] typically having higher HIV prevalence. There are effective interventions to reduce HIV transmission among PWID, and the logic of “combined” prevention programming is that providing multiple interventions on a large scale will lead to greater reductions in HIV infection than can be obtained through providing any single intervention [3]. Whether combined prevention leads to reductions in racial/ethnic and sex disparities in HIV infection remains an open question. A meta-analysis of racial/ethnic disparities [1] found statistically significant disparities at all levels of HIV seroprevalence; significant disparities may exist at low and high HIV prevalence levels. Indeed, it is possible that implementation of combined prevention programming may actually increase relative disparities if the interventions are less accessible to racial/ethnic minority PWID or are less effective for racial/ethnic minority PWID.

New York City has experienced the world’s largest local HIV epidemic among PWID, and racial/ethnic disparities emerged at the start of the epidemic [4]. “Combined prevention” programming for PWID in New York City began with large-scale expansion of syringe exchange programs in the mid-1990s, adding to existing methadone maintenance programs. The numbers of syringes exchanged annually increased from approximately 250,000 in 1990–1993 to approximately 3 million in 2000–2002. This was temporally associated with a reduction in HIV incidence among PWID in the city from 4/100 person-years to 1/100 person-years [5]. Racial/ethnic disparities in HIV infection persisted after the expansion of the syringe exchange programs, however, with adjusted odds ratios of 4.02 for HIV prevalence among African-Americans compared to White PWID and 1.49 for HIV prevalence among Hispanic PWID compared to White PWID, in data collected between 1995 and 2008 [5].

Studies of HIV infection in PWID in Baltimore [6], San Francisco [7] and New York City [8] indicate that sexual transmission has become an increasingly important factor in HIV infection among PWID. In 2007, the New York City Department of Health and Mental Hygiene launched the “New York City Condom Social Marketing Program” [9]. This program includes a specially branded NYC Condom with distinctive packaging and distributes over 30 million free condoms per year [10]. The program reaches drug users through the free condoms distributed at drug treatment and syringe exchange programs, and the program has been generally well-received by drug users in New York City [3, 10].

In this report, we assess whether the implementation of the NYC Condom Social Marketing program, as an additional component of “combined prevention” for HIV, was associated with any change in racial/ethnic disparities in HIV infection among PWID in New York City and the prospects for possible reductions in disparities in the future.

## Methods

The data reported here are derived from ongoing analyses of data collected from drug users entering the Mount Sinai Beth Israel drug detoxification and methadone maintenance programs in New York City. The methods for this “Risk Factors” study have been previously described in detail [5, 11] so only a summary will be presented here. The programs are both large (approximately 5000 admissions per year in the detoxification program and approximately 6000 patients participating in methadone treatment at any point in time) and serve New York City as a whole. There were no changes in the requirements for entrance into the program over the time periods for the data presented here.

Both injecting and non-injecting drug users entering the detoxification and methadone maintenance programs are eligible to participate in the study. Hospital records and the questionnaire results are checked for consistency on route of drug administration and subjects are examined for physical evidence of injecting. The data presented here are from subjects who reported injecting illicit drugs in the 6 months prior to entry into drug treatment and who reported that their first drug injection was in 1995 or later (after large-scale expansion of syringe exchange in New York, “combined prevention”).

In the detoxification program, research staff visited the general admission wards of the program in a preset order and examined all intake records of a specific ward to construct lists of patients admitted within the prior 3 days. All of the patients on the list for the specific ward were then asked to participate in the study. After all of the patients admitted to a specific ward in the 3 day period have been asked to participate and interviews have been conducted among those who agreed to participate, the interviewer moved to the next ward in the preset order. As there was no relationship between the assignment of patients to wards and the order that the staff rotated through the wards, these procedures should produce an unbiased sample of persons entering the detoxification program. In the methadone program, newly admitted patients were asked to participate in the research. In both programs, willingness to participate has been high, with approximately 95% of those asked agreeing to participate.

Written informed consent was obtained and a trained interviewer administered a structured questionnaire covering demographics, drug use, sexual risk behavior, and use of HIV prevention services. Most drug use and HIV risk behavior questions referred to the 6 months prior to the interview.

After completing the interview, each participant was seen by a counselor for HIV and hepatitis C virus (HCV) pretest counseling and serum collection. Participants were tested for HIV and HCV regardless of any previous testing. We did not want to depend upon self-reports of previous testing, and we stored sera for future possible testing. (Storage of sera was included in the informed consent.) HIV testing was conducted at the New York City Department of Health Laboratory using a commercial, enzyme-linked, immunosorbent assays (EIA) test with Western blot confirmation (BioRad Genetic Systems HIV-1-2+0 EIA and HIV-1 Western Blot, BioRad Laboratories, Hercules, CA). Testing for anti-HCV was also conducted by the City Department of Health Laboratory using the Vitros anti-HCV enhanced chemiluminescence immunoassay (Ortho Diagnostics).

Subjects are permitted to participate multiple times in the study, though only once in any one year. We used data from subjects who participated in different years in the analyses presented here, as those subjects were members of the population of interest in the different years. Only about 3% of the subjects in any year were repeat participants, however, so that these subjects do not have a large influence on the results.

HIV counseling and testing has been provided on a large scale for drug users in New York City since the early 1990s. We do not present relationships between risk behaviors in the 6 months prior to the interview and HIV status, as almost all HIV infections would have occurred prior to the 6 month period for recent risk and our preliminary analyses suggest reduction in risk behavior by HIV seropositive persons in order to avoid transmitting HIV to others. Reductions in transmission behavior after HIV testing have been noted in a number of studies [12, 13].

We used the model developed by Vickerman and colleagues [14] to estimate sexual versus injecting related acquisition of HIV among PWID.

We used chi squared tests for comparisons across racial/ethnic groups, and bivariate and multivariable logistic regression analyses to identify factors associated with being HIV seropositive. Stata 12 statistical software [15] was used for statistical analyses. The study was approved by the Mount Sinai Beth Israel Institutional Review Board.

## Results

Table 1 presents demographic characteristics (race/ethnicity, gender and age) and recent drug use and sexual behavior (for the 6 months prior to the interview), date of interview and HIV serostatus by racial/ethnic groups for subjects who began injecting in 1995 or later. There are a number of statistically significant differences by race/ethnicity in the demographic characteristics and recent behaviors. Whites were younger and African-Americans were older; Latino/as were more likely to be male and African-Americans were more likely to be female. Whites were more likely to report injecting cocaine by itself or in combination with heroin (speedball), African-Americans were least likely to report daily injection and most likely to report sniffing heroin and smoking crack cocaine.

**Table 1:** Demographic and drug use characteristics of people with injecting drug use who started injecting in 1995 or later New York City Mount Sinai Beth Israel drug treatment programs.

	Race/Ethnicity			
	White	African-American	Latino/a	Overall
	n (%)	n (%)	n (%)	n (%)
Avg. age (SD)#	32 (0.5)	42 (0.9)	36 (0.4)	35 (0.3)
Total	297 (100)	82 (100)	324 (100)	703 (100)
Males	234 (79)	61 (74)	271 (84)	566 (81)
Injection drugs use				
Speedball	121 (41)	22 (27)	129 (40)	272 (39)
Heroin	284 (96)	77 (94)	310 (96)	671 (95)
Cocaine*	142 (48)	26 (32)	127 (39)	295 (42)
Non-injection drugs use				
Speedball (sniffed)	25 (8)	9 (11)	32 (10)	66 (9)
Speedball (smoked)	19 (6)	7 (9)	16 (5)	42 (6)
Heroin (sniffed)*	127 (43)	64 (78)	146 (45)	337 (48)
Heroin (smoked)	15 (5)	4 (5)	9 (3)	28 (4)
Cocaine (snorted)	73 (25)	20 (24)	74 (23)	167 (24)
Crack Cocaine (smoked)*	127 (43)	50 (61)	112 (35)	289 (41)
Daily injection*	227 (76)	44 (54)	253 (78)	709 (75)
Receptive sharing	70 (24)	16 (20)	72 (22)	158 (22)
Distributive sharing	63 (21)	13 (16)	54 (17)	130 (18)
Unsafe sex w/ primary partner	137 (46)	37 (45)	166 (51)	340 (48)
Unsafe sex w/ casual partner	53 (18)	18 (22)	60 (19)	131 (19)
HIV+*	3 (1)	14 (17)	14 (4)	31 (4)

# Significant difference (p<0.05) across race/ethnicity groups by one-way analysis-of- variance.

* Significant difference (p<0.05) across race/ethnicity groups by chi-square test.

Almost all subjects reported injecting heroin and there were no significant racial/ethnic differences in either “receptive sharing” (injecting with needles and syringes that had been used by others) or “distributive sharing” (passing on needles and syringes that one had used to others).

There were very large differences in HIV serostatus, with 1% of White, 17% of African-American, and 4% of Hispanic subjects testing HIV seropositive. The racial/ethnic group differences in HIV prevalence cannot be explained in terms of recent (past 6 months) injecting risk behaviors, where Whites tended to have the highest rates and African-Americans tended to have the lowest rates of injecting risks. The lack of significant differences in sexual risk behaviors also indicates that recent sexual risk behavior does not explain the substantial differences in HIV prevalence.

We used univariate and multivariable logistic regression to identify factors independently associated with being HIV seropositive. We began with all variables in the univariate analyses and then used backwards elimination to identify factors making independent contribution. Note that all variables that were significant in the univariate analyses remained significant in the multivariable analysis with essentially no change from the univariate to the multivariable (adjusted) odds ratios. Results are presented in Table 2. (As noted in Methods, we did not include recent drug use and recent sexual risk behaviors into these models as our preliminary analyses suggested that HIV positive persons had reduced their risk behaviors.) Gender, race/ethnicity and year of interview were all significantly associated with being HIV seropositive in both univariate analyses and multivariable analyses. The racial/ethnic disparities were large and highly significant, particularly the odds ratios for HIV prevalence among African-American compared to White subjects. HIV prevalence declined over time among these subjects, with an adjusted odds ratio of 0.7 per year.

**Table 2:** Univairate and multivariate logistic models of HIV infection among PWID who began injecting in 1995 or later, and interviewed between 2007- and 2014, New York City Mount Sinai Beth Israel drug treatment programs.

	Univariate		Multivariate	
	OR (95%CI)	95% Confidence Interval	OR (95%CI)	95% Confidence Interval
Race/Ethnicity				
White (ref.)	1.0		1.0	
African- American	20.0	5.6, 72.2	19.0	4.8, 75.8
Latino/a	4.4	1.3, 15.6	4.4	1.2, 15.8
Gender				
Male (ref.)	1.0		1.0	
Female	3.2	1.5, 6.7	3.2	1.4–7.1
Year of interview	0.7	0.6, 0.8	0.7	0.6–0.8
Age	1.1	1.0, 1.1		
Total years since first injection	0.9	0.9, 1.0		

The persistence of the racial/ethnic disparities, particularly the African-American/White disparity, raises questions as to how and when HIV transmission is occurring among these subjects. We used the model developed by Vickerman and colleagues [14] for estimating sexual versus injecting related transmission of HIV among PWID. The HCV/HIV co-infection prevalence among our subjects (prevalence of HCV among HIV positives) was 0.6, and the ratio of HIV prevalence (4%) to HCV prevalence (54%) was 0.074. Applying these data to the model indicates that 75% or more of the HIV infections among these subjects were acquired through sexual transmission.

**Fig 1:**
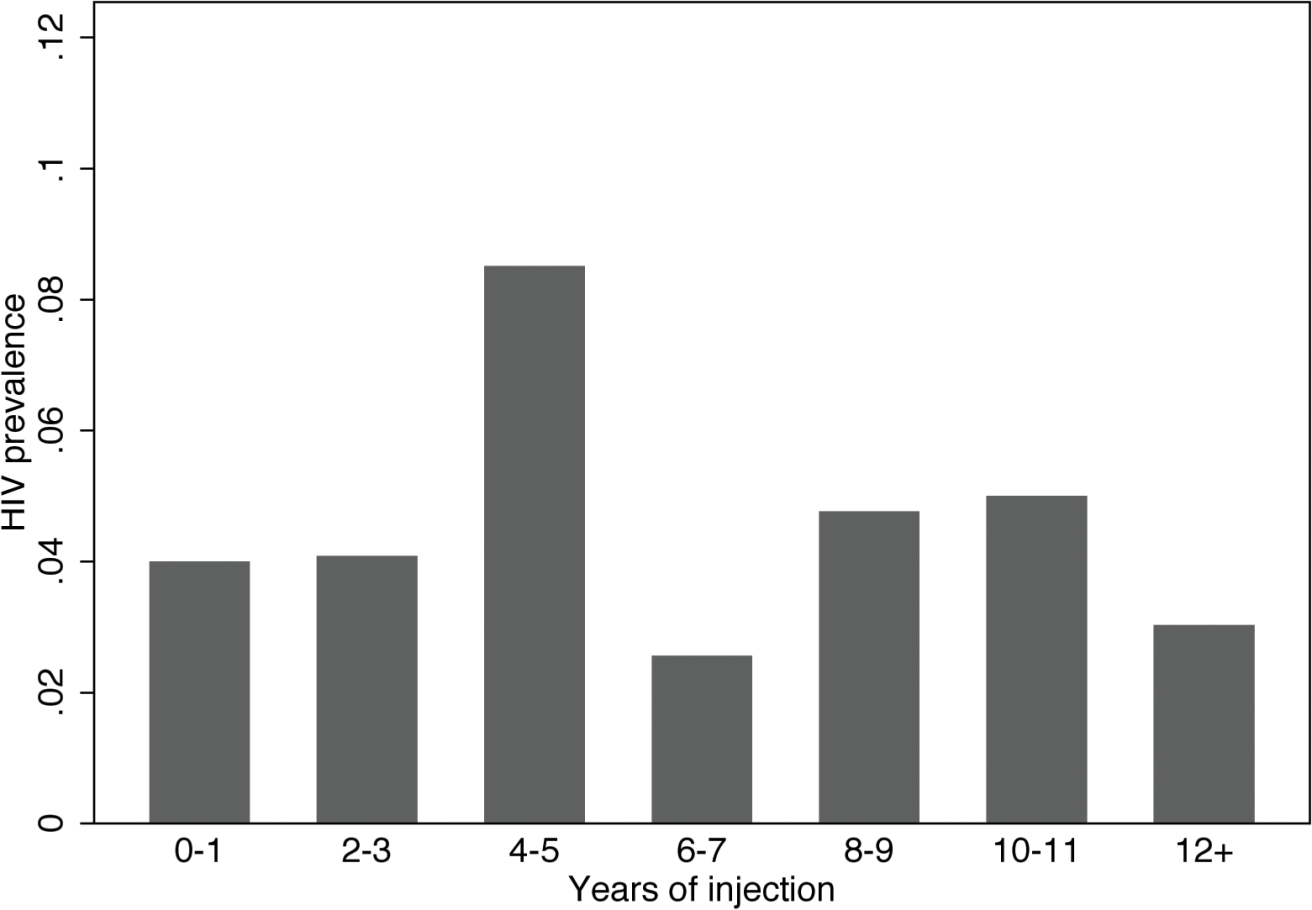
HIV prevalence and years of injection.

We used linear regression to model HIV prevalence as a function of years since first injection among our subjects (Fig 1). This gave:

Likelihood of HIV seropositive=0.04×(0.001)×(yearsofinjecting)

The intercept of 0.04 was highly significant (*P*<0.01), and the slope for years injecting was not significantly different from zero (*P* = 0.5). This linear regression model suggests that a very large percentage of the subjects were HIV seropositive when they began injecting and that very few of the subjects became infected with HIV after they began injecting.

There is additional evidence in our data to support the interpretation that a substantial percentage of the HIV infections occurred prior to first injection. The average age of first drug use (for drugs other than alcohol, nicotine or marijuana) among these subjects was 19, and the average age at first injection was 28, so that the subjects would have had an average of 9 years of using drugs such as heroin, powder cocaine and crack cocaine during which they could have acquired HIV through sexual transmission. Additionally, among the 5 subjects who reported that they were HIV seropositive and had been injecting for 4 years or less at the time of interview, 3 of them reported that they were receiving ART. At the times of interview for these 5 subjects, the New York City guidelines [16] were to begin ART when CD4 cell counts were < 350/mm^3^. Thus, it would appear that these 3 subjects were very likely to have been infected prior to their first injection. (Overall, 35% of subjects who tested HIV positive reported that they were receiving ART at the time of the interview.)

## Discussion

The primary objective of this report was to examine whether “combined prevention” for PWID, including the addition of the NYC Condom Social Marketing program in 2007, was associated with any reductions in racial/ethnic disparities in HIV infection among PWID in New York City. Comparisons of the data reported here with previously published data collected from PWID participating in our research from 1995–2008, however, do not show any reduction in relative disparities. Table 3 presents a comparison of the adjusted odds ratios for racial/ethnic disparities in previously published data collected from 1995–2008 among PWID who began injecting in 1995 or later and interviewed at our research site) [5]. There were no significant differences the 1995–2008 versus the 2007–2014 adjusted odds ratios, note the overlaps in the 95% confidence intervals. The directions of these non-significant changes were towards larger racial/ethnic odds ratio disparities.

**Table 3:** Adjusted odds ratio (aOR) for racial/ethnic disparities in previously published data collected from 1995–2008 among PWID who began injecting in 1995 or later and interviewed at New York City Mount Sinai Beth Israel drug treatment programs.

	1995–2008		2007–2014	
	aOR	95% Confidence Interval	aOR	95% Confidence Interval
Race/Ethnicity				
White (ref.)	1.0		1.0	
African-American	4.02	1.67, 9.69	21.0	5.7, 77.5
Hispanic	1.49	1.02, 2.17	4.5	1.3, 16.3

The current data suggest that the great majority of HIV infections among these subjects occurred through sexual transmission, and that much of this sexual transmission may be occurring prior to first injection (while subjects were using heroin and cocaine through non-injecting routes of administration). Herpes simplex virus type II (HSV-2) infection both facilitates acquisition and transmission of HIV and is strongly associated with HIV infection and racial/ethnic disparities in HIV infection among non-injecting drug users (NIDUs) in New York City [17]. Reduction in racial/ethnic disparities in HIV among PWID may thus require addressing HSV-2 related sexual transmission disparities in HIV infection among NIDUs before they transition to injecting.

Data collection for this report began at the time of implementation of the NYC Condom Social Marketing program. As noted above, this program has generally been well received by injecting and non-injecting drug users in the New York City, and it is possible that the NYC Condom program is contributing to the decline in HIV prevalence that we observed. However, the relative disparities, particularly among African-Americans compared to Whites, have clearly continued. Additional interventions are needed to address these disparities.

**Fig 2:**
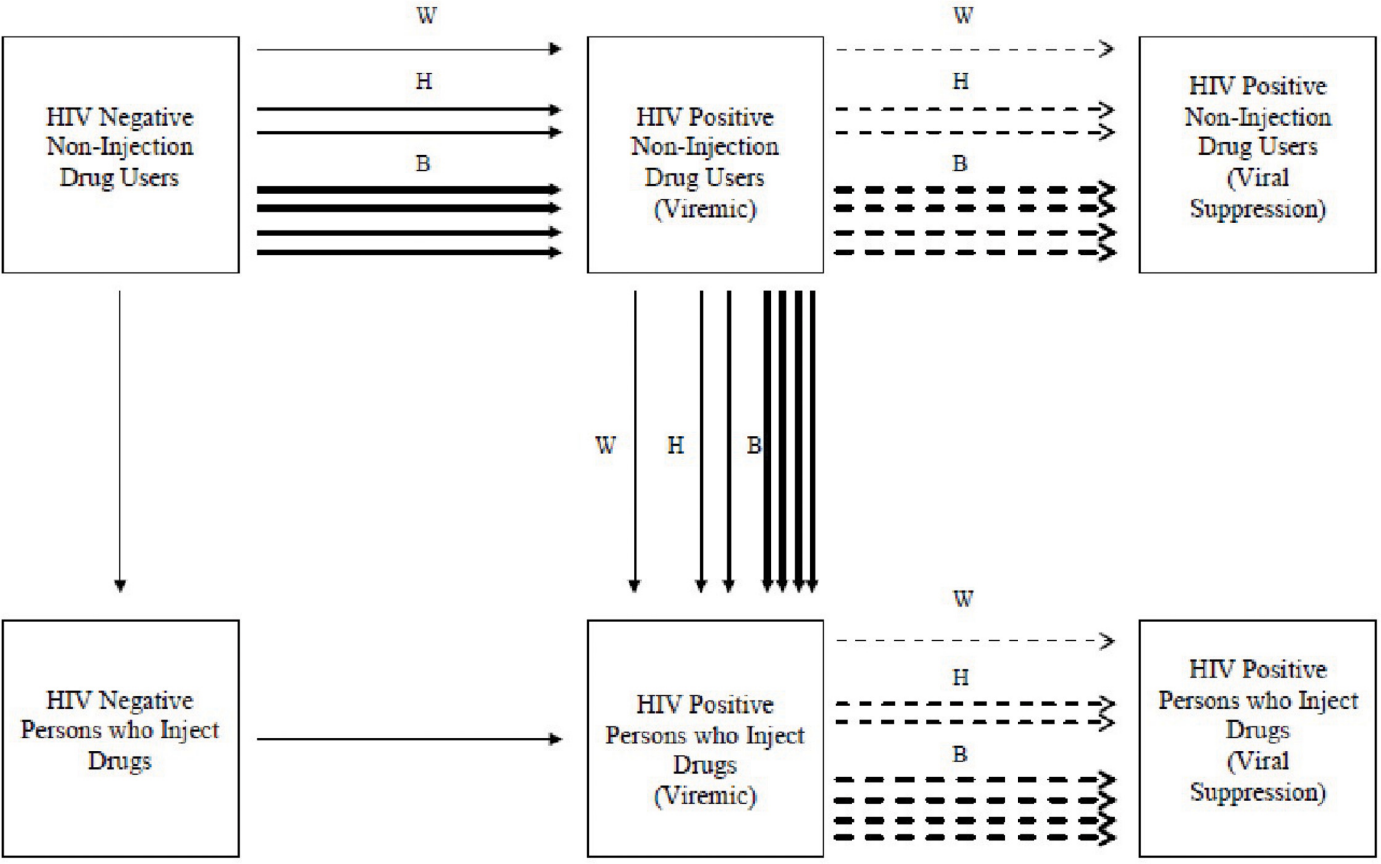
HIV Transmission and Transitions to Injecting Drug Use.

The City Department of Health and Mental Hygiene has developed a policy of offering ART to all HIV seropositives in New York City, with a goal of having 80% or more of HIV seropositives at viral suppression, [18] where they would not be capable of transmitting HIV. This treatment as prevention (TasP) protocol has the potential to successfully address racial/ethnic disparities in HIV among PWID and NIDUs. Fig 2 presents a state transition diagram for transitions from HIV seronegative to HIV seropositive status, from non-injecting to injecting drug use and from HIV seropositive viremic to HIV seropositive viral suppressed status. The numbers of lines for W (White), H (Hispanic) and B (Black) represent the relative proportions of HIV positive drug users among different racial/ethnic groups making the transitions, and thus generating the disparities. For TasP to be effective in eliminating racial/ethnic disparities, it will be necessary to achieve much higher coverage of TasP (dashed lines) among Hispanics and particularly among African-American drug users in New York City. From Table 1, 1% of White PWID are HIV seropositive, and 17% of African-American PWID are HIV seropositive. It would thus require that 16/17 (94%) of HIV seropositive African-American PWID would need to achieve viral suppression just to reach the HIV prevalence among White PWID with 1% of the population is HIV positive.

The CDC has recently recommended large-scale implementation of pre-exposure prophylaxis (PrEP) as another method for reducing HIV transmission in the United States [19]. If PrEP were successfully provided to HSV-2 positive/HIV negative NIDUs and PWID in New York City, it would very likely reduce the existing racial/ethnic disparities. Meaningful reduction in the existing racial/ethnic disparities would, however, require implementing PrEP on a large scale.

In this long-running study, HIV infection among White PWID has almost been eliminated—prevalence among the White PWID in this sample was only 1%. Thus, eliminating the disparities will probably require virtual elimination of new HIV infections among African American and Latino/a drug users. TasP and PrEP have the potential for almost eliminating HIV infection among African American and Latino/a drug users, but will need to be implemented on a large scale to accomplish this.

### Limitations

There are several limitations of this study that should be noted. First, the study design is serial cross sectional surveys, rather than a cohort design. Thus, we do not have data on when HIV seronegative individuals became infected with HIV, and when HIV seropositives might have left the active injecting population due to either cessation of injecting or to disability or death. We also do not have behavior data on HIV seronegatives and HIV seropositives and risk network factors at the actual times of HIV transmission. Thus we cannot directly compare acquisition and transmission risk behaviors and risk network factors among the groups.

An ethically conducted cohort study, however, would have had other important limitations. The repeated contact between research staff and cohort participants would provide many opportunities for positive relationships to develop between staff and participants. These positive relationships should then enhance the effectiveness of the risk reduction counseling that would be provided in an ethically conducted cohort study. The positive relationships with research staff and the repeated risk reduction counseling, along with differential drop out from the cohort, would make the remaining participants less and less representative of the local PWID population.

Also with respect to the date of HIV infections, our data collection period for this report began with the year the Condom Social Marketing program was introduced and then covered the eight years following the introduction. It is possible that there simply were not enough new HIV infections during this time period to create any change in the racial/ethnic disparities that existed prior to the introduction of the Condom Social Marketing program.

Second, even though our comparisons reached conventional levels of statistical significance, we had only modest numbers of HIV seropositive subjects. Larger numbers of HIV seropositive subjects will be needed for analyses of receiving ART over time and of possible racial/ethnic differences in receiving ART.

Third, PWID who enter drug treatment programs may not be representative of all PWID at risk for HIV infection. It is likely that the levels of drug use that would lead to entering drug treatment are associated with higher risk for acquiring HIV infection.

Finally, there was an overlap of two years in the comparison between the new data presented in this report and the previously published data. This is only a modest overlap and would not be expected to obscure any reduction in the racial/ethnic disparities had such a reduction been occurring.

The limitations of the present study are important, but it is difficult to imagine that they would have created the patterns of persistent racial/ethnic disparities. Rather, it would appear that these patterns were observed despite any limitations of the study.

## Conclusions

Racial/ethnic disparities in HIV infection among PWID in New York City have persisted for the last two decades despite successive implementation of additional components of “combined prevention.” It may be helpful to reframe the disparities question to a question of achieving an “AIDS free generation,” [20] in which there are “very few” new HIV infections among adults. The most optimistic findings in our 2007–2014 data are the flat slope of HIV prevalence by years injecting, suggesting very few new HIV infections after beginning to inject, and the reduction in HIV prevalence over the time period (aOR = 0.7/year, 95% CI 0.6–0.8, from Table 2), which does suggest that an “AIDS free” generation may be possible for PWID in New York City. With a current HIV seroprevalence of 1% among White PWID who began injecting in 1995 or later, we may be very close to achieving an AIDS free generation among White PWID in New York City.

Achieving an AIDS free generation for African-American and Hispanic PWID would seem to require considerable additional efforts to reduce sexual transmission among ethnic minority drug users. Treatment as prevention (TasP) and Pre-Exposure Prophylaxis (PrEP) may be important methods for further reducing HIV transmission among PWID and NIDUs. Both TasP and PrEP appear to be very effective in the presence of HSV-2 infection. HIV seropositive drug users who are also HSV-2 seropositive could be given high priority for TasP, and HSV-2 seropositive/HIV seronegative drug users could be given high priority for PrEP. The NYC Condom Social Marketing program should also be continued.

Disparities in HIV infection have persisted since the beginning of the HIV epidemic among PWID in New York City, and now would be the time to increase efforts to finally eliminate these disparities through addressing both injecting and sexual transmission and achieving an AIDS free generation for all groups of injecting and non-injecting drug users in New York City.
